# Clinical validation of automated and rapid mariPOC SARS-CoV-2 antigen test

**DOI:** 10.1038/s41598-021-99886-6

**Published:** 2021-10-13

**Authors:** Juha M. Koskinen, Petri Antikainen, Kristina Hotakainen, Anu Haveri, Niina Ikonen, Carita Savolainen-Kopra, Kati Sundström, Janne O. Koskinen

**Affiliations:** 1 ArcDia International Ltd, Turku, Finland; 2 Faculty of Medicine, University of Turku, Turku, Finland; 3 Mehiläinen Oy, Helsinki, Finland; 4 Department of Clinical Chemistry and Haematology, University of Helsinki, Helsinki, Finland; 5 Finnish Institute for Health and Welfare, Helsinki, Finland; 6 SataDiag, Pori, Finland; 7 Department of Clinical Chemistry, Faculty of Medicine and Health Technology, Tampere University, Tampere, Finland

**Keywords:** Infectious-disease diagnostics, SARS-CoV-2, Viral infection, Nucleoproteins, RNA-binding proteins, Viral proteins, Infection

## Abstract

COVID-19 diagnostics was quickly ramped up worldwide early 2020 based on the detection of viral RNA. However, based on the scientific knowledge for pre-existing coronaviruses, it was expected that the SARS-CoV-2 RNA will be detected from symptomatic and at significant rates also from asymptomatic individuals due to persistence of non-infectious RNA. To increase the efficacy of diagnostics, surveillance, screening and pandemic control, rapid methods, such as antigen tests, are needed for decentralized testing and to assess infectiousness. A novel automated mariPOC SARS-CoV-2 test was developed for the detection of conserved structural viral nucleocapsid proteins. The test utilizes sophisticated optical laser technology for two-photon excitation and individual detection of immunoassay solid-phase particles. We validated the new method against qRT-PCR. Sensitivity of the test was 100.0% (13/13) directly from nasopharyngeal swab specimens and 84.4% (38/45) from swab specimens in undefined transport mediums. Specificity of the test was 100.0% (201/201). The test's limit of detection was 2.7 TCID_50_/test. It showed no cross-reactions. Our study shows that the new test can detect infectious individuals already in 20 min with clinical sensitivity close to qRT-PCR. The mariPOC is a versatile platform for syndromic testing and for high capacity infection control screening of infectious individuals.

## Introduction

Emerging pandemic coronavirus (CoV) was recognized in Wuhan, China, in late 2019. The virus, isolated from patients mentioned to be pneumonic, was quickly sequenced to share 79.6% full length genome similarity with the Severe Acute Respiratory Syndrome virus (SARS-CoV-1) and 91.2% similarity between its nucleocapsid (N) proteins^1^. The novel SARS-CoV-2, causing COVID-19, was identified to be circulating in horseshoe bats for decades similarly to SARS-CoV-1^2^. Diagnostic nucleic acid amplification tests (NAAT), mostly quantitative real-time polymerase chain reaction (qRT-PCR), were quickly developed worldwide, based on protocol provided for World Health Organization^3^. Diagnostic qRT-PCR capacities were ramped up quickly in central laboratories because such tests are fast to develop for new targets. Most often, the new qRT-PCR tests were adopted for clinical diagnostics with minimal verification and validation against other diagnostic test methods.

For the seasonal coronaviruses, the interpretation of gene positivity in clinical specimens has been challenging since the viral RNA is detected at similar rates and qRT-PCR cycle threshold (Ct) values from symptomatic and asymptomatic individuals. The viral RNA is also co-detected with genomes of other respiratory viruses^4–7^. This is also the case for the SARS-CoV-2^8,9^. Moreover, recent scientific evidence indicates that qRT-PCR positivity has poor correlation for assessment of SARS-CoV-2 infectiousness^10–16^. Whereas, Pekosz et al. (2020) showed that the detection of N-protein by an antigen test correlates with SARS-CoV-2 viral culture more accurately than qRT-PCR^13^. Already half a decade ago Inagaki et al. (2016) unequivocally concluded for influenza that, “PCR…is not an appropriate method for indicating infectivity” and “the antigen-detection test estimated the infectious period with comparable if not better accuracy with culture”^17^. In the case of COVID-19 diagnostics, the fact that viral RNA persistence can be detected without viable virus for months, has been a known clinical challenge, as diagnostics relied in the beginning of the pandemic solely on NAAT detection^18^, the efficacy of which is in ruling out positivity.

The expression of N-protein, which is the key pathogenicity factor of coronaviruses^19^, is essential for the coronavirus replication and transcription of the viral RNA^20,21^. Without the accumulation of the N-protein, the coronaviral mRNA is degraded by the nonsense-mediated decay (NMD) pathway of eukaryotic cells^19^. Alexandersen et al. (2020) concluded that the detection of RNA is not an indicator of actively replicating SARS-CoV-2. Their data suggests that virion and subgenomic RNAs are stable in cellular double-membrane vesicles and, therefore, can be detected long after the acute infection^22^. Furthermore, Zhang et al. (2021) found that parts of the reverse-transcribed SARS-CoV-2 RNA can integrate ex vivo into the human genome without the ability to yield infectious viruses and suggest that this could explain at least partly the long term RNA shedding, however, in vivo evidence remains to be seen^23^.

Shortly after viral exposure, viral concentration is low and qRT-PCR Ct values are high. When the virus starts replication, it happens fast. In a cell model, extensive coronavirus RNA transcription has occurred in 6 to 8 h after the infection^24^. In addition, NAATs being prone for reporting clinically insignificant findings (analytically the detection may be correct, there is viral RNA in the sample) they are prone to contaminations. A study of SARS-CoV-2 primer–probe sets from four major European suppliers found a significant level of contamination from the reagents. False positives as low as qRT-PCR Ct 17 were obtained^25^. Low levels of SARS-CoV-2 RNA contamination has also been found from surfaces and air in rooms where mildly ill individuals were isolated without notable viable virus^26,27^. It has also been shown that environmental contamination may yield in positive test results in PCR among individuals sampled in the same area where intranasal influenza vaccine dosing was done^28^. These data suggests that individuals having presence near symptomatic patients can be contaminated by RNA without being infected with viable virus. Thus, methods detecting the viral RNA by amplification are prone for clinically insignificant positive results, especially when significant part of the population has been infected recently. The fact that a positive NAAT result is not a reliable biomarker of active infection or COVID-19, is a true challenge for clinicians and decision making for quarantine. It is not only that a missed necessary quarantine has health and epidemic costs but also that a falsely imposed quarantine has social and financial consequences^29^.

The different performance requirements of diagnostic, surveillance and screening testing have been recently discussed by Mina and Andersen (2020). There is a need for both super sensitive PCR based tests and rapid and appropriately sensitive antigen tests to fight the COVID-19 pandemic^30^. The use of the two methodologies should supplement one another in clinical practice and pandemic fight.

In the present study, we analytically and clinically validated the performance of a novel 2nd generation mariPOC SARS-CoV-2 test (ArcDia International Ltd, Finland), which is a promising test to decentralize and speed up coronavirus testing^31^, as intended for rapid and automated detection of viral acute phase proteins when there is a clinical suspicion of acute COVID-19. Monoclonal antibodies of the test are designed to target a conserved epitope in the N-protein, which is the most abundant protein in coronaviruses. We have previously shown that the presence of coronavirus OC43 N-protein in the nasopharynx correlates with the respiratory tract infection symptoms^32^. It has been shown that clinical presentations of seasonal coronavirus OC43 infections can be similar to those of coronaviruses that are considered as severe viruses (SARS and MERS)^33^.

**Figure 1:**
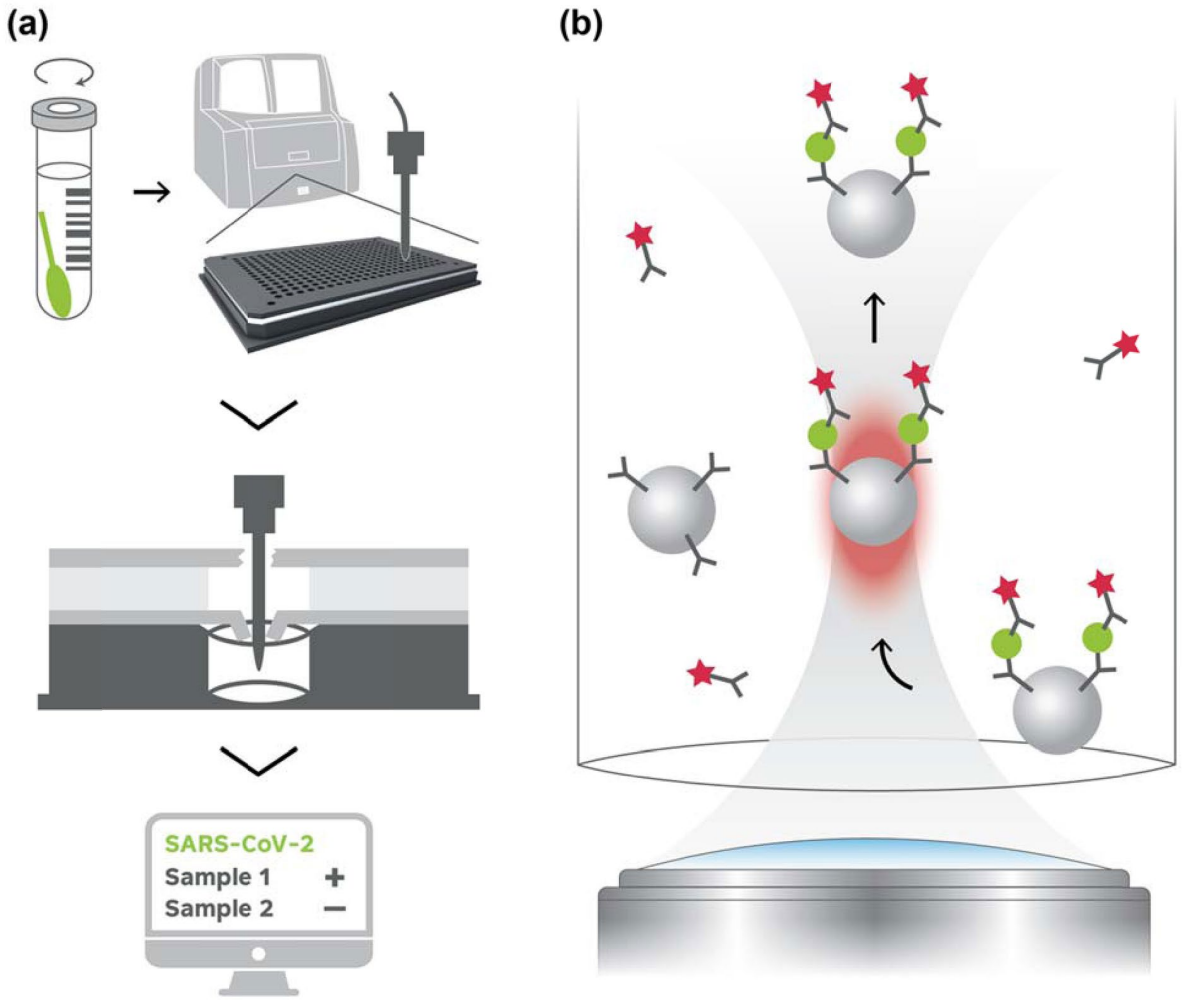
(**a**) Diagnostic workflow in the mariPOC SARS-CoV-2 test and schematic sample dispensing into the test cartridge. Nasopharyngeal sample is suspended into the sample buffer by vortexing before placing the sample tube into the analyzer for automated analysis. The sample is automatically dispensed into the test plate reaction well(s) through resealing multilayer cover, which upper and lower pierceable layers are aluminum foil and cross-cut sheet, respectively. Immunometric reactions start when the sample dissolves the dried reagents. Test result is reported objectively as positive or negative. (**b**) Schematic principle of the SARS-CoV-2 assay where nucleocapsid proteins are detected based on sandwich immunoassay and two-photon excitation fluorescent measurement of individual microparticles by confocal microscopy. Grey hourglass-shaped area designates excitation light bath. Reddish oval-shaped area designates the focal volume where two-photon excitation of fluorescence takes place.

The mariPOC is an automated platform for the rapid multianalyte testing of acute infectious diseases. The mariPOC test’s operational steps, subsequently to nasopharyngeal sampling, are: cutting the swab into sample tube, adding one volume of sample buffer from a bottle-top dispenser, sealing the tube with a piercable cap, vortexing the sample tube in order to release the specimen from the swab, and placing the sample tube into analyzer for automated analysis and objective fluorescent result read out (Fig. 1a). The analyzer aspirates the sample through the piercable cap and dispenses, through resealing multilayer cover, 20 µL aliquots into the reaction chambers (one per tested analyte) containing dried test reagents. Thus, after closing the sample tube cap, the whole analysis is executed without opening any containers having potentially infectious sample. The system has sophisticated autoverification functions to assess the technical reliability of analyses, and the results can be transferred automatically to the laboratory information system and/or as anonymized epidemiological data^34^ into mariCloud™ service. The hands-on time is one minute per sample, and the analyzer works in continuous-feed and walk-away mode. The mariPOC SARS-CoV-2 test is available as a single pathogen test and as part of syndromic multianalyte tests covering, among others, influenza viruses. The throughput of one mariPOC analyzer is up to 300 single analyte tests or 100 multianalyte tests in 24 h. The results are reported in two phases, great majority of the infectious cases in twenty minutes and very low positive and negative cases in 55 min or two hours, depending on the test configuration.

The automated platform is based on a separation-free two-photon excitation assay technique (Fig. 1b). In the two-photon excited fluorescence assay technique, bioaffinity assay and signal detection are performed inside microvolume reaction chambers of 384 well format cartridge in one step, without physically separating the bound and the unbound fractions of target analytes and the reagents. Instead, the separation is brought about by optical phenomena^35,36^. Target analytes (here SARS-CoV-2 nucleocapsid proteins) are captured from the sample with specific monoclonal antibodies onto the surface of solid-phase carrier polystyrene microparticles (Fig. 1b). When fluorescent monoclonal antibody conjugates (tracer)^37^ bind to the captured antigens, three component immunocomplexes are formed directly and quantitatively in proportion to the concentration of the analyte in the sample. The fluorescent brightness of individual microparticles is measured, one by one, by scanning through the transparent bottom of the 384 well plate with a focused laser beam (1064 nm). The beam is deflected using piezo-driven mirrors. A microparticle entering the focus backscatters the excitation light and the microparticle is pushed by optical forces through the focus, which is similar in size to that of the microparticle. The two-photon excited fluorescence brightness of the particles at visible wavelengths is measured during the backscattering. The fluorescence is also measured from the solution phase when there is no particle in the focus. The ratio of apparent brightness of the microparticle to solution signal (unbound tracer and sample matrix) depends on the degree of bioaffinity binding. In the absence of binding, the ratio is close to unity. Data reduction algorithms calculate the mean brightness of the particles and the solution phase, and compare it to preset cut-off to determine the qualitative or quantitative result reported to the user on the graphical user interface^35,36^.

## Results

### Analytical sensitivity

We evaluated the Limit of Detection (LoD) of the mariPOC SARS-CoV-2 test as 50% tissue culture infective dose (TCID_50_) for the concentration that gives ≥ 95% positivity for the replicates analyzed. The LoD was 2.7 TCID_50_/test in 20 µL reaction volume for gamma-irradiation inactivated culture supernatant, at which all twenty replicates gave a positive test result. Based on the certificate of analysis of the viral preparation, the LoD equals to 1690 genome equivalents per test. Similarly studied LoD, as q-RT PCR Ct value, was 33 for UV-inactivated SARS-CoV-2 culture supernatant.

### Cross-reactivity

Analytical specificity of the mariPOC test was studied by challenging the test against relevant microbes commonly found in the nasal cavity. The microbe stocks were suspended into mariPOC RTI sample buffer and analyzed. The test gave negative result with seasonal coronaviruses (OC43, 229E, or NL63) and other tested microbes, but it gave a positive test result for recombinant N-protein of SARS-CoV-1 (Table 1).

**Table 1:** Cross-reaction study information and results (+ or −). Viruses were purified viral culture preparations (concentration) or supernatants (dilution) and bacteria were culture suspensions inactivated by heating. Bacterial concentration of 4 × 10^7^ bct/mL (OD600 = 0.04) in reaction was based on that OD600 = 1.0 corresponds to 10^9^ bct/mL.

Analyzed microbe	Source	Reaction concentration	Result
Human coronavirus OC43 ATCC VR1558	ATCC	5 × dilution	−
Human coronavirus 229E ATCC VR740			−
Human coronavirus NL63	Academic Medical Center, Amsterdam, the Netherlands		−
SARS coronavirus 1 (purified nucleoprotein)	ArcDia International Ltd, Finland	1 µg/mL	+
MERS coronavirus (purified nucleoprotein)		1 µg/mL	−
Human coronavirus HKU1 (purified nucleoprotein)		10 µg/mL	−
Influenza A virus H3N2 A/Panama/2007/99	Hytest Ltd, Turku, Finland	5 µg/mL	−
Influenza A virus H3N A/Texas/50/12	Research Institute of Influenza, St Petersburg, Russia	10 µg/mL	−
Influenza A virus H3N2 A/Victoria/361/11		10 µg/mL	−
Influenza B virus Phuket/3073/2013		100 × dilution	−
Influenza A virus H1N1 A/New Caledonia/20/99	Biomarket Ltd, Turku Finland	5 µg/mL	−
Influenza A virus (so-called swine flu) H1N1v A/FIN/554/09	Finnish institute for health and welfare	40 × dilution	−
Respiratory syncytial virus type A / Long	AbD Serotec Inc, now Bio-Rad Laboratories, Inc	7.4 × dilution	−
Respiratory syncytial virus type B / clinical strain (EQAS round 1, 2017)	Labquality Ltd, Helsinki, Finland	2 × dilution	−
Human metapneumovirus	Department of Virology, University of Turku, Finland	10 × dilution	−
Parainfluenza 2 virus		25 × dilution	−
Human bocavirus (clinical specimen)	Turku University Hospital, Finland	Nasopharyngeal swab suspended in 1.3 mL	−
Human bocavirus (purified VP2 antigen)	Vilnius University, Institute of Biotechnology, Lithuania	5 µg/mL	−
Parainfluenza 1 virus Sendai	Hytest Ltd, Turku, Finland	50 µg/mL	−
Parainfluenza 3 virus Washington/1957 C243	AbD Serotec Inc, now Bio-Rad Laboratories, Inc	20 µg/mL	−
Adenovirus strain 6	Hytest Ltd, Turku, Finland	4 µg/mL	−
*Streptococcus pneumoniae* ATCC 49619	Finnish institute for health and welfare	4 × 10^7^ bacteria/mL	−
*Streptococcus pyogenes* ATCC 19615			−
*Staphylococcus aureus* ATCC 29213			−
*Staphylococcus epidermidis (isolate)*			−
*Streptococcus anginosus* (209)			−
*Streptococcus constellatus* (5690			−
*Streptococcus intermedius (1343)*			−
*Haemophilus influenzae* ATCC 33391			−
*Haemophilus parainfluenzae* ATCC 33392			−

### Clinical specificity

Diagnostic specificity of the mariPOC test was validated by analyzing 205 freshly sampled nasopharyngeal swabs according to the protocol shown in Fig. 1a. Two of the samples were positive in both the mariPOC test and routine PCR test. Three and two of the sample analyses were rejected by the autoverification in the preliminary and final result reporting phases giving failure rates of 1.5% and 1.0%, respectively. Rest of the samples was negative. Thus, the specificity and positive predictive value of the test was 100.0% in the preliminary (200/200) and final (201/201) result reporting phases.

### Clinical sensitivity

We validated sensitivity of the mariPOC SARS-CoV-2 test with 58 frozen qRT-PCR positive nasopharyngeal samples from two specimen cohorts. The sensitivity of the test was 100.0% (13/13) in the preliminary and final result reporting phases in sensitivity cohort 1, where the nasopharyngeal swab specimens were suspended directly into the mariPOC sample buffer or first into saline (Tables 2 and 3). Prevalence of SARS-CoV-2 in the first sample cohort was 6%, which is well in alignment with the prevalence during the study time in the geographical area (5%).

**Table 2:** Sensitivity (positive percent agreement) of the mariPOC SARS-CoV-2 test when compared with the qRT-PCR methods.

Sample cohort	mariPOC result phase	No. of specimens		Sensitivity (%) (95% CI)
		TP	FN	
1	Final	13	0	100.0 (75.3–100.0)
	Preliminary	13	0	100.0 (75.3–100.0)
2	Final	38	7	84.4 (70.5–93.5)
	Preliminary	33	12	73.3 (58.1–85.4)

TP = true positive, FN = false negative, CI = confidence interval (exact Clopper-Pearson method).

**Table 3:** mariPOC and qRT-PCR SARS-CoV-2 results (+ or −) with Ct values that were available for the sensitivity cohort 1. Comparator qRT-PCR was not done for the sample number 106 from unknown reason but was considered to have been positive as it would have been analysed because of antigen and confirmatory qRT-PCR positivity (Ct values for genes: E, 30.2; RdRP, 35.2; N, 35.78).

Sample		mariPOC result		Comparator qRT-PCR (Gene, Ct value)
Number	Type	Preliminary	Final	
5	Dry swab	**+**	**+**	**+**(NA)
7	Dry swab	**+**	**+**	**+**(NA)
13	Dry swab	**+**	**+**	**+**(NA)
21	Dry swab	**+**	**+**	**+**(NA)
47	Dry swab	**+**	**+**	**+**(NA)
62	Dry swab	**+**	**+**	**+**(NA)
95	Swab in saline	**+**	**+**	**+**(NA)
106	Swab in saline	**+**	**+**	Considered as + (NA)
151	Dry swab	**+**	**+**	**+**(NA)
198	Swab in saline	**+**	**+**	**+**(N, 21.89; ORF1ab, 20.66)
202	Swab in saline	**+**	**+**	**+**(N, 23.58; ORF1ab, 21.8)
203	Swab in saline	**+**	**+**	**+**(N, 28.82; ORF1ab, 26.5)
206	Swab in saline	**+**	**+**	**+**(N, 24.41; ORF1ab, 24.15)

NA = not available/applicable.

**Figure 2:**
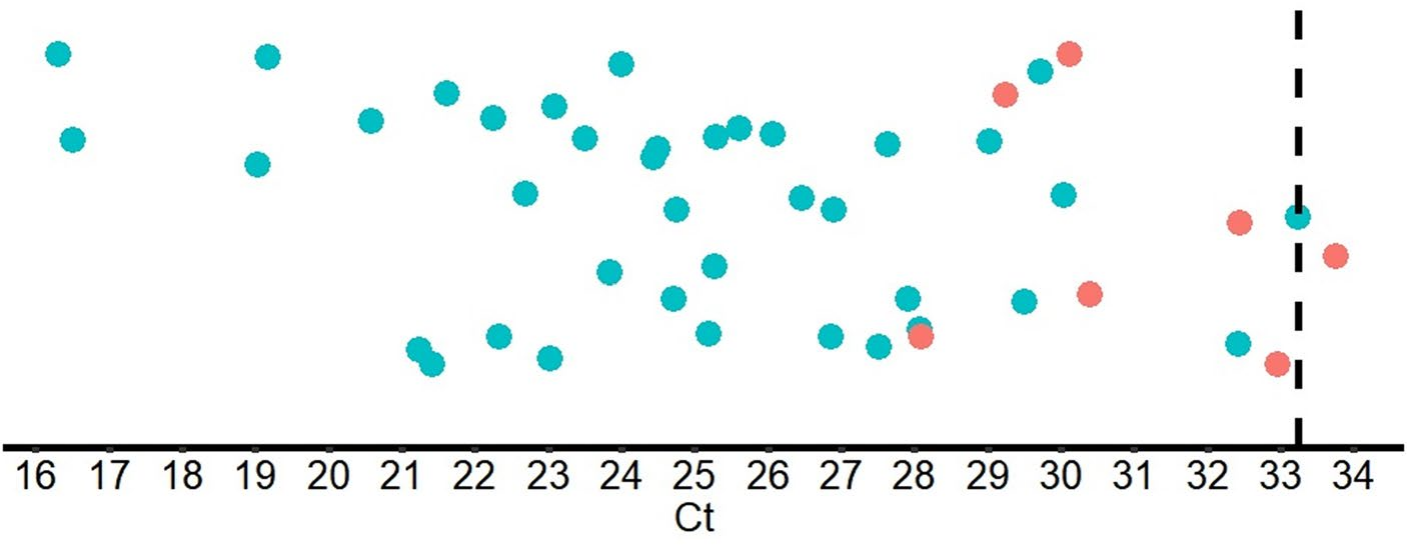
Ct values of qRT-PCR for mariPOC test positive (green dots) and negative (red dots) samples in the validation sample cohort 2. Dashed line is at Ct 33.24, which was the lowest detected Ct.

The sensitivity of the test in sensitivity cohort 2 was 73.3% (33/45) and 84.4% (38/45) in the preliminary and final result reporting phases, respectively, when the nasopharyngeal swabs were initially suspended in undefined transport mediums and further diluted with the mariPOC sample buffer (Table 2). Based on 95% confidence interval, both cohorts had similar statistical reliability (Table 2). Overall, 38 out of 45 samples were positive with the mariPOC in sensitivity cohort 2 (Fig. 2). The test showed 100% (31/31) positivity rate compared to qRT-PCR for Ct values ≤ 28 (Table 4). Above the Ct value 28, the positivity rate of the mariPOC declined as typical for an antigen test, reaching 91.9% (34/37) with Ct values ≤ 30.

**Table 4:** Comparison of cumulative positivity rates of viral culture (four studies) and mariPOC (sensitivity sample cohort 2) to qRT-PCR below different Ct categories^10,12,14,48^.

	Arons (%)	La Scola (%)	Singanayagam (%)	Basile (%)	mariPOC	
					Preliminary	Final
Ct < 25	87	83	85	89	100.0% (20/20)	100.0% (20/20)
Ct < 28	77	77	NA	86	90.3% (28/31)	100.0% (31/31)
Ct < 30	67	75	74	82	83.8% (31/37)	91.9% (34/37)
Overall	62	68	30	NA	73.3% (33/45)	84.4% (38/45)

NA = not applicable.

## Discussion

When setting up a diagnostic process or choosing a diagnostic method, one should carefully consider, to start with, whether the disease, clinical condition and use case, require high sensitivity for ruling out or high specificity for ruling in. There is a need for both in fighting the COVID-19 pandemic. In general, analytically highly sensitive testing, such as PCR testing, is good at ruling out a disease (e.g. keeping a ward clean) while highly specific testing, such as antigen testing, is good at ruling in a disease (e.g. acute infection diagnostics and assessing infectiousness). Because of rapidity and lesser logistic challenges compared to central lab testing, antigen testing is particularly good in surveillance, field-testing, screening of masses, cohorting of inpatients, acute disease diagnostics, and in assessing the infectiousness of individuals^13,38^. Especially when disease prevalence is low, clinical specificity of the screening and diagnostic testing should be emphasized to keep unnecessary quarantines and economic damages at minimum while still allowing sufficient enough infection control^30^.

According to scientific data, to effectively prevent spread of the disease, pandemic control should prioritize accessibility, frequency of testing, and rapid sample-to-answer time over test sensitivity^39–41^. Viral load and probability to infect others is highest just prior to onset of symptoms and during the symptomatic phase^42^.

We described here analytical and clinical validation of mariPOC SARS-CoV-2 test sensitivity (equals to positive percent agreement in this study) and specificity. Determination of LoD was performed with gamma-irradiation inactivated viral culture supernatant and showed that only less than three infectious units per test was needed for positive test result. LoD was also determined as qRT-PCR Ct units using UV-inactivated virus. The obtained Ct LoD was 33, which approaches the theoretical analytical sensitivity of a typical PCR method with 5 µl cDNA volume and applying 95% confidence interval. The maximum Ct values detected in clinical samples (Fig. 2 and Table 3) were similar to the determined Ct LoD.

Based on high identity (89.1%) between SARS-CoV-1 (Uniprot entry, P59595) and SARS-CoV-2 (UniProt entry, P0DTC9) nucleocapsid protein sequences, and obtaining positive result for purified SARS-CoV-1 nucleocapsid protein in cross-reactivity testing (Table 1), it is likely that the mariPOC test detects also the SARS-CoV-1 virus itself. Cross-reactions were not observed. A minor limitation of the study is that cross-reactivity for MERS coronavirus and coronavirus HKU1 were assessed using purified protein (Table 1) and not with clinical samples or cultured virus. However, it is unlikely that the mariPOC test would cross-react with MERS or HKU1 as they share only 44.2% (UniProt entry, K9N4V7) and 28.9% identity (UniProt entry, Q5MQC6), respectively, in their nucleocapsid protein peptide sequence with the SARS-CoV-2.

Our sensitivity validation cohort 1 showed 100% sensitivity. While the highest qRT-PCR Ct obtained by the primary reference test in this cohort was 30.2, the cohort consisted of unselected and consecutive samples collected from patients with clear symptoms. This might explain why there were no samples with higher Ct values. The sensitivity cohort 2 showed 100% positivity rate for the mariPOC below qRT-PCR Ct 28 (Table 2 and Fig. 2). This result was excellent taking into account that the samples were unfavorable for the mariPOC platform with separation-free fluorescent measurement. Colorful transport media are not recommended for mariPOC testing since they elevate fluorescent signal levels^43^ and unnecessarily dilute the samples, which reduces sensitivity. The pooled sensitivity of sample cohorts 1 and 2 at qRT-PCR Ct ≤ 30 was 94%, suggesting even higher sensitivity for mariPOC compared to what has been reported in the literature for SARS-CoV-2 viral culture against qRT-PCR, as summarized in Table 4. In addition, our results are in line with at least two other N-protein detecting tests that were evaluated against RT-PCR and culture^44,45^. Several studies have shown that infectivity of SARS-CoV-2 declines rapidly in samples showing qRT-PCR Ct above 25, and viable virus is rarely isolated after 8 days from onset of the symptoms. The detection of sole viral RNA, especially at low levels without the detectable level of viral N-protein or culture positivity, is a questionable marker of acute infection and infectiousness^10–14,16,46–48^.

The results suggest that the clinical sensitivity of the mariPOC test (84.4% in unfavorable sample matrix to 100.0% when used according to manufacturer recommendations) is similar or even better than that of at least some rapid RT-PCR tests (93.4%)^41^, when symptomatic patients suspected with acute COVID-19 infection are tested within the first five days of symptoms and prevalence among tested samples is reasonable (6% in sensitivity cohort 1). Recommended sample in the mariPOC test is native nasopharyngeal swab specimen suspended into 1.3 mL of the RTI sample buffer. Other specimen types may yield in lower apparent sensitivity. In the sensitivity cohort 1, suspending part of the swabs first into saline prior to the addition of mariPOC RTI sample buffer followed by a further dilution into mariPOC RTI sample buffer by a factor of two for the testing, diluted the specimens 4 to 20 times (2 to 4.3 PCR Ct units) more than the recommended sample pretreatment. Additional dilution lowers the sensitivity compared to the recommended protocol, and could have led to an underestimation of the test sensitivity.

Strengths of the sensitivity validation included that the specimens were collected in the early phase of the COVID-19 pandemic in Finland that minimized the detection of RNA persistence with the RT-PCR among the cohort population.

Limitations of the validation study were that the patient characteristics and the number of symptomatic days before sampling were not available for the study. Freezing and thawing of the positive samples prior to mariPOC testing and additional dilutions to the recommended protocol were also limitations. However, if any, these could have had a negative effect on the mariPOC test sensitivity and, hence, the study at least did not overestimate the sensitivity of the mariPOC SARS-CoV-2 test.

## Conclusions

The mariPOC SARS-CoV-2 test is an automated, highly specific and clinically accurate test with rapid sample-to-answer time for individuals with clinical suspicion and in acute phase of an infection. In comparison to other antigen detection tests, such as lateral flow assays, the closed tube test system and the design of operational steps minimize specimen handling and possible exposure of user to infectious material. The multianalyte syndromic tests help to differentiate between SARS-CoV-2 and other viruses, such as influenza. The single analyte test provides high capacity of 300 samples a day at the point-of-sampling. Objective result read-out and LIS connectability minimize manual work and human errors. Our study together with other scientific data suggests that the mariPOC can detect majority of the cases already in 20 min with sensitivity similar to a rapid PCR while maximum sensitivity is achieved in 55 min. The positivity rate of mariPOC compared to qRT-PCR Ct values in clinical samples is very high (> 90%) up to Ct 28–30, and samples at least up to Ct 33.24 (Fig. 2) or 35.78 (Table 3) can be detected depending on the PCR method and gene target. The detection of conserved epitope in the N-protein of SARS coronaviruses with the mariPOC likely provides accurate information about infectiousness similarly to other antigen tests and viral culture and suggests ability to detect also emerging virus variants. Further studies using viral culture as comparative method and follow-up of infectiousness of patients using antigen detection are needed in order to optimize viral respiratory tract infection management.

## Materials and methods

### Analytical sensitivity

Nasopharyngeal swab specimens from asymptomatic individuals were pooled and suspended into mariPOC RTI sample buffer (B02, ArcDia International Ltd) into volume corresponding to 1.3 mL per swab. This pooled clinical sample matrix (1.3 mL) was spiked with 75 µL of gamma-irradiation inactivated SARS-CoV-2 cell lysate (stock 2.8 × 10^6^ TCID_50_/mL, USA-WA1/2020, NR-52287, lot 70035888, BEI Resources, Manassas, VA, USA) in culture supernatant of different viral concentrations. The samples were analyzed with the mariPOC SARS-CoV-2 test following the manufacturer’s instructions. LoD was determined as the lowest concentration giving at least 19 positives out of 20 replicates (≥ 95% positivity). LoD as Ct value was determined similarly for UV-inactivated SARS-CoV-2 culture supernatant (2 × 10^6^ PFU/mL, Ct 17, University of Helsinki, Finland)^45^. Based on available information (material safety data sheet), the RTI sample buffer is Tris-based (< 1.0%) buffer (pH 8) containing surfactants, bovine serum albumin and sodium azide (< 0.1%).

### Cross-reactivity

Analytical specificity of the mariPOC SARS-CoV-2 test was studied by challenging the test against relevant microbes commonly found in the nasal cavity (see Table 1 for species, strains and titers). Briefly, the microbe stocks were suspended in the mariPOC RTI sample buffer and analyzed with the mariPOC test.

### Clinical specificity

Validation of the specificity of the mariPOC SARS-CoV-2 test was conducted in SataDiag laboratory unit in Pori, Finland in February 2021 by one operator following the manufacturer’s instructions. In the study, 205 freshly sampled nasopharyngeal swab specimens were analyzed. The samples were leftover samples from routine diagnostics with the 1st generation (launched May 2020) mariPOC SARS-CoV-2 test. Samples positive in mariPOC were also analyzed with the Xpert Xpress SARS-CoV-2 test (ref XPRSARS-COV2-10) detecting N2 and E-genes, Cepheid, USA.

### Clinical sensitivity

Sensitivity of the mariPOC SARS-CoV-2 test was validated with 58 frozen qRT-PCR positive nasopharyngeal samples from two specimen cohorts.

#### Sample cohort 1

The first cohort consisted of 13 qRT-PCR positive nasopharyngeal swab samples collected from patients (N = 211) visiting primary healthcare COVID-19 drive-in stations of Mehiläinen Oy in Helsinki capital area of Finland from March to April 2020. The qRT-PCR negative frozen samples were not analyzed for this study because the test specificity was studied with fresh samples as described above. The enrollment criteria were respiratory infection symptoms and clinician’s suspicion of COVID-19, the official criteria for COVID-19-testing in Finland, and at the clinical study sites at the time of the study. The samples were taken with a flocked swab from the nasopharynx (8 to 12 cm deep for adults and 4 to 8 cm deep for children) by rotating the swab in nasopharyngeal cavity for 10 s. Two consecutive specimens were collected from 127 patients. The specimen for standard of care testing was collected first. These specimens were analyzed after RNA extraction with Allplex™ 2019-nCoV RT-PCR assay (Seegene Inc., Republic of Korea) at Seoul Clinical laboratories (Republic of Korea). Allplex™ 2019-nCoV RT-PCR assay detects E, N and RdRP genes^49^. The second swab specimen was kept in a dry tube at + 4 °C for a maximum of 8 h and stored frozen until analysis with the mariPOC SARS-CoV-2 test. For 84 patients, one nasopharyngeal swab specimen was collected. These swabs were suspended into saline (0.5 − 1 mL) and analyzed with Amplidiag COVID-19 qRT-PCR assay including RNA extraction (Mobidiag Ltd, Finland) at Vita Laboratorio Ltd (Finland). Amplidiag COVID-19 qRT-PCR assay detects N and ORF1ab genes^41^. The leftover saline specimens were stored frozen until analysis with the mariPOC SARS-CoV-2 test.

The dry swab specimens and leftover saline samples were analyzed retrospectively with the mariPOC test by two operators following the manufacturer’s instructions. Briefly, dry swabs were suspended into 1.3 mL of the mariPOC RTI sample buffer and the leftover saline samples (range 0.1 − 0.65 mL) were diluted with mariPOC RTI sample buffer to a final volume of 1.3 mL. For this validation study, the samples were further diluted into the mariPOC RTI sample buffer in one-to-one (1:1) ratio and analyzed with the mariPOC test. When a discrepant result between the mariPOC and comparator RT-PCR was obtained, the swab samples taken for the mariPOC were confirmatory tested at the Department of Clinical Microbiology, Turku University Hospital (Finland) with an in-house reference qRT-PCR detecting E, N and RdRP genes^3^.

#### Sample cohort 2

The second cohort consisted of forty five positive pseudonymized specimens with known qRT-PCR Ct values (16 to 34) from the frozen nasopharyngeal swab specimen library of Finnish Institute of Health and Welfare, Helsinki, Finland, in undefined transport mediums. The qRT-PCR protocol was an in-house method based on the primers and probes by Corman et al. (2020) ^3^. The cohort consisted mostly of symptomatic, but in part also of asymptomatic subjects, while the detailed information for each subject was not available for this study. The specimens were in either reddish (N = 22) or colorless (N = 23) solutions. The samples were diluted into the mariPOC RTI sample buffer in one-to-one (1:1) ratio and analyzed with the mariPOC test. The positivity rate of the mariPOC test was evaluated against different qRT-PCR Ct value categories and then compared to published viral culture studies.

### Ethical approval

The specimens were collected during an internal laboratory method validation study. The study was not linked with treatment of patients. The samples were not individually identifiable and they were either leftover samples or samples collected in parallel to routine diagnostics after having obtained informed consent. The study was approved by ethics committees of Mehiläinen Oy and SataDiag laboratory division, and was performed in accordance with the relevant guidelines and regulations.

## Data Availability

Line data is available upon request.
